# Surface-EMG Amplitude Differences Before and After Scheduled University Team Training Following a Standardized Warm-Up in Male Collegiate Volleyball Players

**DOI:** 10.3390/jcm15166460

**Published:** 2026-08-20

**Authors:** Maria Migdał, Magdalena Wiacek, Rafał Studnicki

**Affiliations:** 1 Student Scientific Circle of Orthopedic Manual Therapy, Medical University of Gdańsk, 80-211 Gdańsk, Poland; m.migdal@gumed.edu.pl; 2 Faculty of Medical Sciences and Health Sciences, University of Radom, Chrobrego 27, 26-600 Radom, Poland; m.wiacek@urad.edu.pl; 3 Department of Physiotherapy, Medical University of Gdańsk, Ul. Dębinki 7, 80-211 Gdańsk, Poland

**Keywords:** surface electromyography, RMS amplitude, shoulder-girdle muscles, collegiate volleyball, repeated measures, training-session assessment

## Abstract

**Objective**: To examine acute BEFORE–AFTER changes in maximum, mean, and median surface-EMG RMS amplitudes recorded from four shoulder-girdle muscles around scheduled collegiate volleyball training sessions. **Methods**: Sixteen male collegiate volleyball players representing one university team participated in this exploratory prospective repeated-measures study. Surface EMG was recorded from the upper trapezius, lower trapezius, infraspinatus, and serratus anterior during muscle-specific maximal voluntary isometric effort tasks. A standardized dynamic warm-up was completed before the BEFORE assessment. Surface EMG was then recorded immediately after warm-up and before the volleyball-specific training components of the session (BEFORE), and again within 5–10 min after completion of the scheduled team-training session (AFTER), on three weekly measurement days. For each EMG outcome, the main inferential estimand was the marginal AFTER–BEFORE contrast averaged across muscles and measurement days. Linear mixed-effects models included assessment condition, muscle, measurement day, and the assessment condition × muscle term; interaction analyses were treated as exploratory because the participant-level sample comprised 16 independent experimental units. Marginal AFTER–BEFORE contrasts were adjusted across the three RMS-amplitude outcomes using the Bonferroni method. **Results**: Mean RMS amplitude was lower AFTER than BEFORE the training sessions by 4.16 µV (95% CI: −6.32 to −2.01; Bonferroni-adjusted *p* < 0.001). Maximum RMS amplitude was also lower by 5.77 µV (95% CI: −11.19 to −0.35), but this contrast was not supported after multiplicity adjustment (adjusted *p* = 0.115). Median RMS amplitude showed an estimated increase of 3.88 µV (95% CI: −0.19 to 7.94; adjusted *p* = 0.181). Exploratory assessment condition × muscle interactions were not statistically significant for any of the three RMS-amplitude outcomes. **Conclusions**: Only the decrease in mean RMS amplitude remained statistically supported after adjustment across the three EMG outcomes. Because both assessments were performed after the standardized warm-up, warm-up status was held constant with respect to completion of the warm-up. The AFTER assessment additionally followed the subsequent volleyball-specific training components and a 5–10-min post-session interval. Accordingly, the contrast represents a within-session AFTER–BEFORE difference associated with the intervening training exposure and temporal/order effects rather than an isolated causal effect of training. Exploratory condition × muscle interactions were not statistically significant, but the small sample does not establish equivalent responses among muscles. The findings do not establish altered mechanical force or localized neuromuscular fatigue.

## 1. Introduction

Volleyball exposes the shoulder complex to repeated overhead actions, particularly serving and spiking, which are central technical components of match and training performance [1]. Biomechanical analyses of volleyball serving and spiking show that these actions place substantial demands on the glenohumeral joint, the rotator cuff, and the scapular stabilizers [1]. Repeated volleyball-specific loading has also been associated with shoulder overuse problems in volleyball players [2]. During these movements, effective shoulder function depends on coordinated activation of the rotator cuff, deltoid, trapezius, and serratus anterior muscles to maintain glenohumeral stability, scapular control, and efficient kinetic-chain force transfer [3,4,5,6]. Repeated volleyball-specific loading may therefore contribute not only to structural overload but also to transient alterations in neuromuscular control that can occur before pain, injury, or measurable performance decline [7,8].

Shoulder problems are clinically and performance-relevant in volleyball, with risk influenced by competitive level, exposure context, and accumulated overhead load [6,9,10]. Common shoulder conditions in volleyball and other overhead sports include rotator cuff tendinopathy, subacromial impingement, bursitis, and altered scapular mechanics [10,11,12]. Reported risk factors include shoulder strength imbalance, reduced glenohumeral range of motion, external rotator weakness, scapular dyskinesis, and impaired neuromuscular control [3,5,13,14,15]. These factors are particularly relevant in volleyball because repeated serving, attacking, blocking, and defensive actions require rapid coordination between scapular stabilizers and glenohumeral muscles [16,17,18,19]. Accordingly, assessing acute neuromuscular responses in the shoulder during volleyball-specific training may provide useful insights into early functional changes in real-world sport conditions [20,21,22].

Surface electromyography provides a practical method for examining training-related changes in shoulder muscle activation. In overhead athletes, EMG-derived measures can help characterize alterations in motor-unit recruitment, activation amplitude, and muscle coordination during or after fatiguing activity [23,24,25,26].

Maximum RMS amplitude represented the highest value of the RMS amplitude envelope within the selected contraction interval. Mean RMS amplitude summarized the average electrical amplitude during that interval, whereas median RMS amplitude provided a complementary distributional summary that was less influenced by brief high-amplitude portions of the signal. These variables were surface-EMG amplitude measures; mechanical force and joint torque were not recorded, and none of the three measures was treated as a stand-alone index of neuromuscular fatigue.

Neuromuscular fatigue is generally defined as a transient reduction in the capacity to generate force or maintain the required level of muscle activation and may result from both central and peripheral mechanisms [27,28]. In EMG-based assessments, fatigue-related responses may be reflected by changes in signal amplitude or spectral properties, including altered recruitment, firing behavior, synchronization, conduction velocity, or metabolic state [27,28]. Nevertheless, amplitude-derived EMG indices should be interpreted as indicators of altered neuromuscular state rather than as definitive evidence of peripheral fatigue, particularly when spectral fatigue indices, such as median or mean frequency, are not analyzed [27,28,29].

Previous studies examining shoulder fatigue and neuromuscular function in overhead athletes have often used isolated laboratory protocols, including repeated external rotation tasks, controlled fatigue procedures, or sport-specific tasks performed outside regular training [30,31,32]. Although such studies provide important mechanistic insight, these protocols do not fully reproduce the intermittent, multidirectional, and technically complex demands of volleyball training. In addition, available EMG and shoulder-function studies have frequently included heterogeneous populations, such as baseball players, swimmers, handball players, clinical cohorts, surgical patients, or rehabilitation populations, rather than collegiate volleyball players assessed during regular team practice [30,31,32,33,34,35]. Therefore, the applicability of existing findings to male collegiate volleyball players assessed during structured university-team preparatory training remains uncertain.

From a training-load perspective, the scheduled volleyball session represented a common external exposure, whereas BEFORE–AFTER differences in surface-EMG amplitude described short-term changes in the recorded signal [7].

However, because individual external and internal training loads were not quantified, the present measurements cannot establish a dose–response relationship between training load and the recorded EMG-amplitude differences, nor can mechanical performance be inferred because force and joint torque were not measured.

Therefore, the main objective of this exploratory study was to estimate the overall marginal AFTER–BEFORE difference in maximum, mean, and median surface EMG RMS amplitudes, averaged across the four examined muscles and three measurement days. Secondary exploratory analyses assessed whether the magnitude of the AFTER–BEFORE difference varied among muscles or measurement days. We expected an overall difference between the BEFORE and AFTER assessments in at least one RMS-amplitude outcome; no directional muscle-specific hypothesis was specified. Nonsignificant interaction tests were not interpreted as evidence of equivalent responses among muscles.

## 2. Materials and Methods

### 2.1. Study Design

This exploratory study used a prospective repeated-measures observational design. On each of the three measurement days, all participants first completed the same standardized dynamic warm-up. The first surface-EMG assessment (BEFORE) was performed immediately after completion of the warm-up and before the subsequent volleyball-specific technical, tactical, and game-based training components. The second assessment (AFTER) was performed within 5–10 min after completion of the scheduled team-training session. Thus, both BEFORE and AFTER measurements were obtained after the standardized warm-up.

The surface-EMG assessment procedure was identical at the two time points. The same body positions, muscle-specific maximal isometric effort tasks, joint angles, stabilization procedures, investigator-applied resistance, verbal instructions, electrode configuration, acquisition settings, trial duration, recovery intervals, and signal-processing procedures were used during BEFORE and AFTER. Therefore, the two assessments were directly analogous with respect to both the EMG measurement protocol and completion of the standardized warm-up.

The experimental contexts nevertheless differed because the AFTER assessment additionally followed the volleyball-specific training components of the scheduled session and a 5–10-min post-session interval. Accordingly, the AFTER–BEFORE contrast represents the within-session difference observed across the intervening volleyball-specific training exposure. Potential effects of elapsed time, repeated testing, measurement order, and early recovery cannot be separated from that training exposure because no non-training time-control condition was included.

Because the BEFORE–AFTER sequence was determined by the temporal organization of the scheduled training session, randomization of assessment order was not applicable. Each participant contributed paired repeated observations under the same standardized measurement procedure and after the same warm-up protocol.

### 2.2. Participants

Participants were recruited through direct invitation by the training staff; consequently, this was a convenience sample drawn from a single university team. The study included 16 male collegiate volleyball players from that team who were preparing for the Polish Academic Volleyball Championships. The sample characteristics were as follows: age, 20.81 ± 1.63 years; height, 187.69 ± 6.96 cm; and body mass, 84.31 ± 9.24 kg. Playing positions included outside hitters (n = 5), middle blockers (n = 5), setters (n = 4), and liberos (n = 2).

No a priori sample-size calculation was performed. The analytical sample comprised all 16 eligible players from the participating university team who completed the required assessments. Although repeated measurements generated multiple observations per participant, the number of independent experimental units remained 16; repeated observations therefore did not compensate fully for the limited participant-level sample size. The study may consequently have had insufficient power to detect small condition × muscle, condition × day, or higher-order interaction effects. All interaction analyses were therefore considered exploratory, and nonsignificant results were interpreted as an absence of statistically detectable evidence in this sample rather than as evidence that the corresponding effect was absent.

The inclusion criteria were active participation in the university volleyball team preparing for the Polish Academic Volleyball Championships, regular attendance at team training sessions, and age ≥ 18 years. The participant-classification framework proposed by McKay et al. [36] was considered; however, participation in a national academic championship alone did not establish that each player met the individual performance criteria required for Tier 3 classification. The sample is therefore described as male collegiate volleyball players rather than as national-level, elite, or international-level athletes.

All participants were in good general health and had no diagnosed musculoskeletal or systemic disease. Exclusion criteria included injury or illness during the study period, chronic upper-limb pain at baseline, glenohumeral joint instability or excessive mobility, neurological disorders, and connective tissue disorders.

The Independent Bioethics Committee for Scientific Research at the Medical University of Gdańsk, Poland, reviewed and approved the study protocol (resolution no. KB/95-242/2025, 10 April 2025). All procedures were conducted in accordance with the Declaration of Helsinki. Before participation, all players received detailed verbal and written information regarding the purpose, procedures, and potential risks of the study. We obtained written informed consent from all participants. Participation was voluntary, and all personal data was handled confidentially.

### 2.3. Training Context and Measurement Schedule

Measurements were performed during a three-week preparatory period preceding the Polish Academic Volleyball Championships. This period represented a structured university-level volleyball mesocycle, with weekly exposure comprising approximately 6 h of volleyball-specific training and 1 competitive match. Accordingly, the investigated training context should be interpreted as university-level collegiate volleyball rather than professional or international elite volleyball. Consequently, neither the magnitude of the individual training stimulus nor between-player differences in the observed EMG-amplitude changes could be related to playing position, action volume, or an objectively or subjectively quantified training dose.

The weekly microcycle followed a consistent structure throughout the three-week observation period. The main training session and experimental measurements were conducted on Wednesday between approximately 17:00 and 19:00. Thursday and Friday were devoted to lower-intensity technical and tactical training; Saturday included a competitive match; Sunday and Monday were scheduled as recovery or rest days; and Tuesday comprised tactical preparation and video analysis. Thus, each Wednesday measurement session was preceded by the Tuesday tactical preparation and followed by lower-intensity technical and tactical sessions before the subsequent competitive match. No additional strenuous upper-body training was scheduled immediately before the Wednesday measurement session. The same general microcycle structure was maintained during all three measurement weeks.

Experimental measurements were performed once per week during the principal structured training session of the weekly microcycle. Each scheduled session lasted approximately 120 min and comprised a standardized dynamic warm-up followed by volleyball-specific technical drills, tactical exercises, and game-based play. On each measurement day, participants completed the standardized warm-up before the BEFORE EMG assessment. The BEFORE assessment was performed immediately after warm-up and before the subsequent volleyball-specific components of the session. Following completion of those components, the AFTER assessment commenced within 5–10 min. Thus, the sequence was: standardized warm-up → BEFORE EMG assessment → volleyball-specific technical, tactical, and game-based training → AFTER EMG assessment. The EMG assessment protocol itself was unchanged between BEFORE and AFTER. Although all participants followed the same session plan, individual exposure could differ according to playing position and participation in specific drills. Counts of jumps, serves, attacks, and blocks; accelerometer- or player-tracking-derived load; position-specific exposure; session rating of perceived exertion; and subjective fatigue were not recorded. Consequently, individual external and internal training loads could not be quantified, and participation in the same scheduled session should not be interpreted as equivalent neuromuscular loading.

### 2.4. Muscle-Specific Maximal Voluntary Isometric Effort Tasks for EMG Recording

Maximal voluntary isometric effort testing was conducted with participants seated upright, with the trunk maintained in a neutral position and both feet placed flat on the floor. Unless otherwise specified, the back was unsupported. The assessment comprised four separate muscle-specific isometric tasks—one for each examined muscle—rather than a single general shoulder task. Each task used a predefined joint position, stabilization procedure, and direction of manually applied resistance. All measurements were performed on the right shoulder, and the same side was tested during every assessment.

The infraspinatus was assessed with the upper arm adducted against the trunk at 0° of shoulder abduction and the elbow flexed to 90°. A towel roll approximately 2–3 cm thick was positioned between the elbow and the trunk to standardize humeral alignment and limit compensatory deltoid involvement. The forearm was initially maintained in a neutral position. The investigator stabilized the elbow against the trunk and applied medially directed resistance to the distal forearm immediately proximal to the wrist, perpendicular to the forearm. Participants performed maximal isometric external rotation while maintaining humeral position.

For upper trapezius testing, participants performed maximal scapular elevation while avoiding lateral flexion of the cervical spine. Inferiorly directed resistance was applied over the acromion, opposing the direction of scapular elevation. Participants were instructed to sustain the elevated scapular position against maximal manual resistance.

The lower trapezius was tested with the participant’s trunk supported against the backrest to restrict lumbar extension and thoracic compensation. The tested arm was elevated to approximately 135° in the scapular plane, corresponding to a seated Y position, with the elbow fully extended and the humerus externally rotated so that the thumb pointed upward. Participants were instructed to combine scapular depression and retraction while maintaining the prescribed arm position. The investigator applied resistance to the distal forearm immediately proximal to the wrist. Resistance was directed inferiorly and anteriorly within the scapular plane. Participants sustained the standardized position during maximal isometric effort.

For serratus anterior testing, participants remained seated with the trunk supported against the backrest in a neutral upright position to limit thoracic flexion and rotation. The tested shoulder was flexed to 90° in the scapular plane, approximately 30° anterior to the frontal plane, with the elbow fully extended and the forearm in neutral rotation. From this position, participants were instructed to protract the scapula by reaching forward without flexing the elbow or altering the shoulder-elevation angle. The investigator stabilized the contralateral shoulder girdle and trunk to restrict trunk rotation, thoracic flexion, and compensatory horizontal adduction or elevation of the tested shoulder. Posteriorly directed resistance was applied to the distal forearm immediately proximal to the wrist, along its longitudinal axis and opposite to the direction of protraction. Participants maintained maximal scapular protraction without elbow flexion, trunk displacement, or shoulder elevation.

Because all maximal isometric effort trials involved static muscle actions, neither muscle-shortening nor muscle-lengthening velocity was present, and no range of motion was evaluated. Real-time visual feedback from the MyoResearch 2.8 software (Noraxon, Scottsdale, AZ, USA) was available throughout testing. The same investigator administered standardized, strong verbal encouragement during every trial to facilitate maximal and consistent effort. Three maximal isometric effort trials were completed for each muscle, with 20 s of recovery between successive trials to minimize fatigue-related carryover.

All maximal isometric effort trials were performed against manual resistance provided by the investigator; neither a dynamometer nor a fixed-resistance apparatus was used, and mechanical force and joint torque were not recorded. The investigator progressively increased resistance until the participant could no longer produce visible movement and then maintained the limb in the prescribed position. Participants were instructed to produce maximal voluntary isometric effort without altering the target joint position. The same investigator conducted all tests using standardized hand placement, stabilization, resistance direction, and verbal encouragement.

The same body position, joint angles, stabilization procedure, sensor location, and verbal instructions were used during all BEFORE and AFTER assessments and on all three measurement days. The highest maximum RMS EMG amplitude obtained across the three trials was used for analysis, and EMG variables were extracted from the corresponding contraction window.

Each maximal isometric effort trial lasted 3 s. This brief duration was selected to obtain a maximal isometric effort while limiting fatigue-related carryover across the three trials performed for each of the four muscles. The duration was a study-specific procedural choice rather than a separately validated diagnostic protocol for shoulder fatigue. We analyzed the complete 3-s interval, including contraction onset and release. RMS amplitude was calculated using a 300-ms sliding window. Maximum RMS amplitude was defined as the highest RMS-window value, mean RMS amplitude as the arithmetic mean of all RMS-window values, and median RMS amplitude as the 50th percentile of these values. Median RMS amplitude was retained as a complementary exploratory amplitude-distribution summary and not as an independently validated physiological fatigue endpoint. No frequency-domain fatigue analysis was performed, and the 3-s contractions were not used to estimate within-trial changes in median or mean frequency.

### 2.5. Surface Electromyography Recordings

Surface electromyography signals included in the final analysis were recorded from four shoulder-girdle muscles: upper trapezius (TU), lower trapezius (TL), infraspinatus (IF), and serratus anterior (SA). These muscles were selected for their functional relevance to scapular control, glenohumeral stabilization, and overhead volleyball actions, as well as their suitability for repeated surface EMG assessment. The subscapularis was not assessed because it is a deep muscle and is not reliably accessible using surface electrodes.

**Figure 1:**
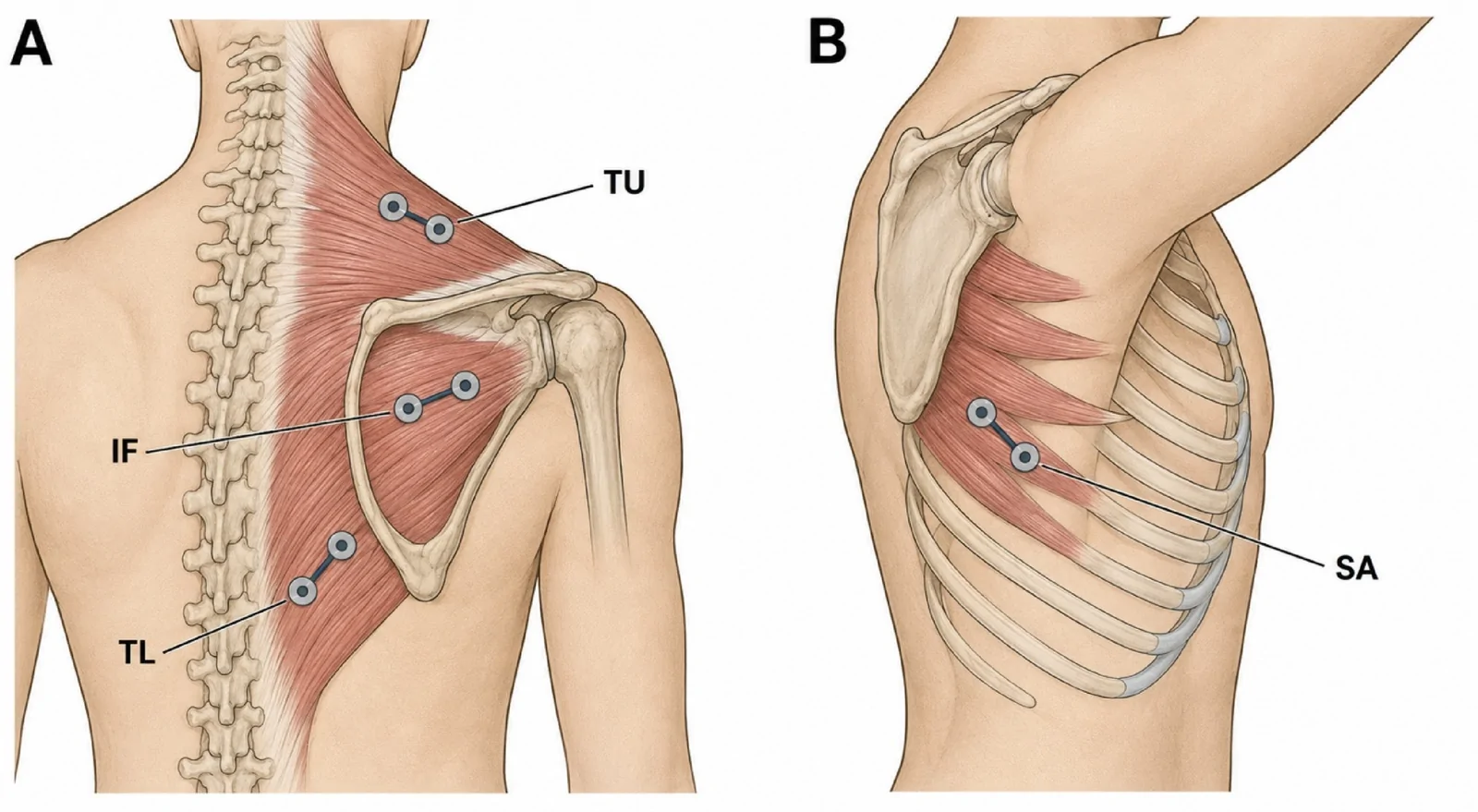
Representative surface-electrode placement for the examined shoulder-girdle muscles. (**A**) Posterior view showing bipolar electrode placement over the upper trapezius (TU), lower trapezius (TL), and infraspinatus (IF). (**B**) Lateral view showing electrode placement over the serratus anterior (SA). Electrode pairs were positioned over the muscle bellies and aligned parallel to the presumed direction of the underlying muscle fibers. The anatomical illustration is schematic and was created using an artificial-intelligence (AI)-assisted image-generation tool for illustrative purposes. The authors reviewed the final illustration to ensure that the depicted anatomical landmarks and electrode locations were consistent with the experimental protocol. The image does not represent an individual study participant or participant-derived data and is intended solely to clarify electrode-placement landmarks and support reproducibility of the surface-electromyography protocol.

Electrode placement (Figure 1) followed established surface EMG recommendations [37]. Before electrode placement, the skin was shaved when necessary, gently abraded, and cleaned with alcohol. Pairs of Ag/AgCl surface electrodes were placed over the muscle bellies and aligned parallel to the presumed direction of the muscle fibers. The center-to-center interelectrode distance was 20 mm. Electrodes were positioned as follows: upper trapezius, midway between the C7 spinous process and the acromion; lower trapezius, along the line between the medial scapular spine and the lower thoracic spine; infraspinatus, within the infraspinous fossa below the scapular spine; and serratus anterior, on the lateral thoracic wall along the rib fibers, anterior to the latissimus dorsi. Within each measurement day, the same electrodes and electrode positions were used for the BEFORE and AFTER recordings; electrodes were not removed or repositioned between the two assessments. Placement was performed by the same researcher using predefined anatomical landmarks.

Because measurements were separated by one week, electrodes were removed after each session and reapplied at the subsequent weekly assessment. To reduce between-week repositioning variability, the same anatomical landmarks, 20-mm center-to-center spacing, fiber orientation, trained investigator, and standardized verification contractions were used at every session. Nevertheless, residual variability due to electrode repositioning cannot be excluded [38]. The EMG analyst was not blinded to BEFORE/AFTER labels; however, signal processing used the same predefined settings for all recordings.

Surface EMG data were collected using a TeleMyo DTS system (Noraxon, Scottsdale, AZ, USA) and 1 cm^2^ Ag/AgCl surface electrodes (Sorimex, Toruń, Poland). Signals were differentially amplified with a gain of ×500, band-pass-filtered between 15 and 500 Hz, and sampled at 1500 Hz with 16-bit resolution. Data were archived and analyzed using MyoResearch 2.8 software (Noraxon).

Signal processing included full-wave rectification and calculation of root-mean-square EMG amplitude using a 300-ms sliding window. Mean, maximum, and median RMS amplitude values were calculated from the smoothed EMG signal and expressed in µV. Surface EMG quantified the electrical activity detected over the examined muscles and was not treated as a direct measure of muscle force. No mechanical force or joint-torque outcome was recorded or analyzed.

Because EMG amplitudes were analyzed as absolute RMS values rather than normalized amplitudes, between-muscle comparisons were interpreted cautiously. Consequently, muscle was retained as a factor in the statistical model to evaluate patterns within the repeated-measures design, but differences in absolute RMS magnitude between muscles were not interpreted as physiological differences in activation level. Normalization is important when comparing EMG amplitude across muscles, participants, or repeated electrode placements [29]. Accordingly, the main inferential focus was placed on model-estimated within-participant BEFORE–AFTER differences rather than on direct comparisons of absolute amplitude between muscles. Nevertheless, a repeated-measures design does not eliminate possible within-session recording variability caused by perspiration, electrode movement, changes in skin–electrode impedance, or other alterations in recording conditions.

Accordingly, the observed BEFORE–AFTER differences cannot be unequivocally attributed to physiological changes in muscle activation because changes in recording conditions during the intervening volleyball-specific training period.

### 2.6. Statistical Methods

All analyses were conducted in R version 4.5.0 using the readxl, dplyr, tidyr, lme4, lmerTest, broom, mixed, emmeans, performance, and ggplot2 packages. Before analysis, we screened the data for completeness and structural integrity by checking missing values, duplicate observations, and the expected number of measurements per participant.

The primary inferential estimand for each EMG outcome was the marginal AFTER–BEFORE contrast averaged over muscles and measurement days. The assessment condition × muscle interaction and all higher-order interaction analyses were treated as exploratory tests of heterogeneity and were not used to infer equivalence among muscles.

Assessment condition was a two-level categorical factor identifying the BEFORE and AFTER measurements. Maximum, mean, and median RMS amplitude were retained as distinct summaries of the distribution of RMS-window values within the analyzed 3-s contraction. Maximum RMS represented the single highest RMS-window value, mean RMS represented the arithmetic average across all RMS windows, and median RMS represented the central value of the RMS-window distribution and was therefore less influenced by isolated high-amplitude windows than the arithmetic mean. Median RMS was included only as a complementary distributional amplitude summary and was not considered a spectral measure, a substitute for median frequency, or an independently validated marker of fatigue. Median RMS amplitude was retained as a complementary distributional summary of the RMS-window values because it is less influenced by isolated high-amplitude windows than the arithmetic mean. It was not assigned an independent physiological interpretation and was not treated as a substitute for a spectral fatigue measure.

Separate main linear mixed-effects models were fitted for each EMG outcome. Within each model, the main inferential estimand was the marginal AFTER–BEFORE contrast averaged equally across muscles and measurement days. The assessment condition × muscle term was retained to explore heterogeneity of the AFTER–BEFORE contrast among muscles; this interaction analysis was exploratory and was not a test of physiological equivalence. Given the participant-level sample of 16, all condition × muscle, condition × day, and higher-order interaction analyses were interpreted as exploratory. Participant identity was included as the grouping factor. We first evaluated a correlated participant-specific random intercept and condition slope. In case this specification was singular or produced a convergence warning, we evaluated an uncorrelated random-slope model. A random-intercept-only model was used as the final fallback when both random-slope specifications were singular or non-convergent.

All categorical predictors used sum-to-zero contrasts. We evaluated fixed effects using Type III tests with Satterthwaite-adjusted degrees of freedom. F statistics, numerator and denominator degrees of freedom, *p*-values, and partial eta-squared values were reported. Marginal and conditional R^2^ values were calculated to describe the variance explained by the fixed effects and by the complete model, respectively. Convergence status, singularity, selected random-effects structures, and random-effect estimates are reported in Supplementary Table S1.

Marginal AFTER–BEFORE contrasts were estimated from each primary model and averaged equally over muscles and measurement days. Estimates are reported with standard errors, 95% confidence intervals, unadjusted *p*-values, and Bonferroni-adjusted *p*-values. Bonferroni adjustment was applied across the three analysed RMS-amplitude outcomes, corresponding to an adjusted interpretive threshold of α = 0.0167 [39].

Two model-specification sensitivity analyses were performed. First, we compared the primary parsimonious model with a random-intercept-only specification. Second, we fitted an exploratory full model containing assessment condition × muscle × measurement day using the same random-effects selection hierarchy. Marginal contrasts from the exploratory full models are reported in Supplementary Table S2, and comparisons across the primary, random-intercept-only, and full three-way models are presented in Supplementary Table S3.

All numerical outputs included in the manuscript and Supplementary Material, including descriptive statistics, mixed-effects model estimates, analysis-of-variance tables, estimated marginal means, and post hoc comparisons where applicable, were exported programmatically to support transparent reporting and reproducibility.

## 3. Results

The primary inferential results were the model-estimated marginal AFTER–BEFORE contrasts. Mean RMS amplitude was 4.16 µV lower at the AFTER assessment than at the BEFORE assessment (SE = 1.10 µV, 95% CI −6.32 to −2.01; Bonferroni-adjusted *p* < 0.001). This was the only one of the three RMS-amplitude contrasts supported after multiplicity adjustment. Maximum RMS amplitude was estimated to be 5.77 µV lower (95% CI −11.19 to −0.35; adjusted *p* = 0.115), whereas median RMS amplitude was estimated to be 3.88 µV higher (95% CI −0.19 to 7.94; adjusted *p* = 0.181).

Exploratory assessment condition × muscle tests did not provide statistically detectable evidence that the BEFORE–AFTER difference varied among muscles for maximum RMS amplitude (*p* = 0.549), mean RMS amplitude (*p* = 0.731), or median RMS amplitude (*p* = 0.137). These nonsignificant interactions were not interpreted as evidence of equivalent muscle responses.

### 3.1. Descriptive Statistics

For descriptive purposes, observations were pooled across the three measurement days. Therefore, each muscle–condition combination included up to 48 observations, corresponding to 16 participants assessed across three weekly sessions. Descriptive statistics for maximum, mean, and median RMS amplitudes, and median root-mean-square electromyographic amplitude are presented by muscle and assessment condition in Table 1.

For mean RMS amplitude, pooled BEFORE values ranged from 79.18 ± 12.01 µV in the upper trapezius to 84.84 ± 15.09 µV in the serratus anterior, whereas pooled AFTER values ranged from 76.61 ± 7.57 µV in the upper trapezius to 81.04 ± 5.23 µV in the serratus anterior. For maximum RMS amplitude, pooled BEFORE values ranged from 95.28 ± 18.10 to 102.75 ± 18.69 µV, whereas pooled AFTER values ranged from 90.91 ± 17.56 to 94.94 ± 19.30 µV. For median RMS amplitude, pooled BEFORE values ranged from 51.10 ± 11.88 µV in the serratus anterior to 74.27 ± 16.04 µV in the upper trapezius, whereas pooled AFTER values ranged from 59.06 ± 24.06 µV in the serratus anterior to 77.08 ± 14.00 µV in the upper trapezius.

Because RMS amplitudes were expressed in absolute units, between-muscle differences are reported as model-derived statistical effects but are not interpreted as evidence that one muscle exhibited greater physiological activation than another. The principal comparisons of interest were the within-participant BEFORE–AFTER effects and whether these changes varied statistically by muscle or measurement day.

### 3.2. Mixed-Effects Model Results

We fitted separate primary linear mixed-effects models for maximum RMS amplitude, mean RMS amplitude, and median RMS amplitude. We selected a correlated participant-specific condition slope for maximum and median RMS amplitudes. For mean RMS amplitude, both correlated and uncorrelated random-slope specifications were singular; consequently, the non-singular random-intercept model was selected. All selected primary models converged without singularity (Supplementary Table S1). Marginal and conditional R^2^ values were 0.051 and 0.332 for maximum RMS amplitude, 0.069 and 0.082 for mean RMS amplitude, and 0.254 and 0.426 for median RMS amplitude, respectively.

Overall BEFORE–AFTER conclusions were based on model-estimated marginal contrasts averaged equally across muscles and measurement days, rather than on any single reference-level or coded fixed-effect coefficient.

Type III fixed-effect tests from the primary models are presented in Table 2. These omnibus tests are reported as supporting model information; the principal inferential results are the model-estimated marginal AFTER–BEFORE contrasts presented in Supplementary Table S2. Because EMG amplitudes were not normalized, muscle main effects are not interpreted as physiological differences in activation among the examined muscles.

Estimates are marginal contrasts derived from the primary mixed-effects models and averaged equally over muscles and measurement days. Positive values indicate higher amplitude AFTER the training session. We applied a Bonferroni adjustment across the three primary EMG outcomes.

**Figure 2:**
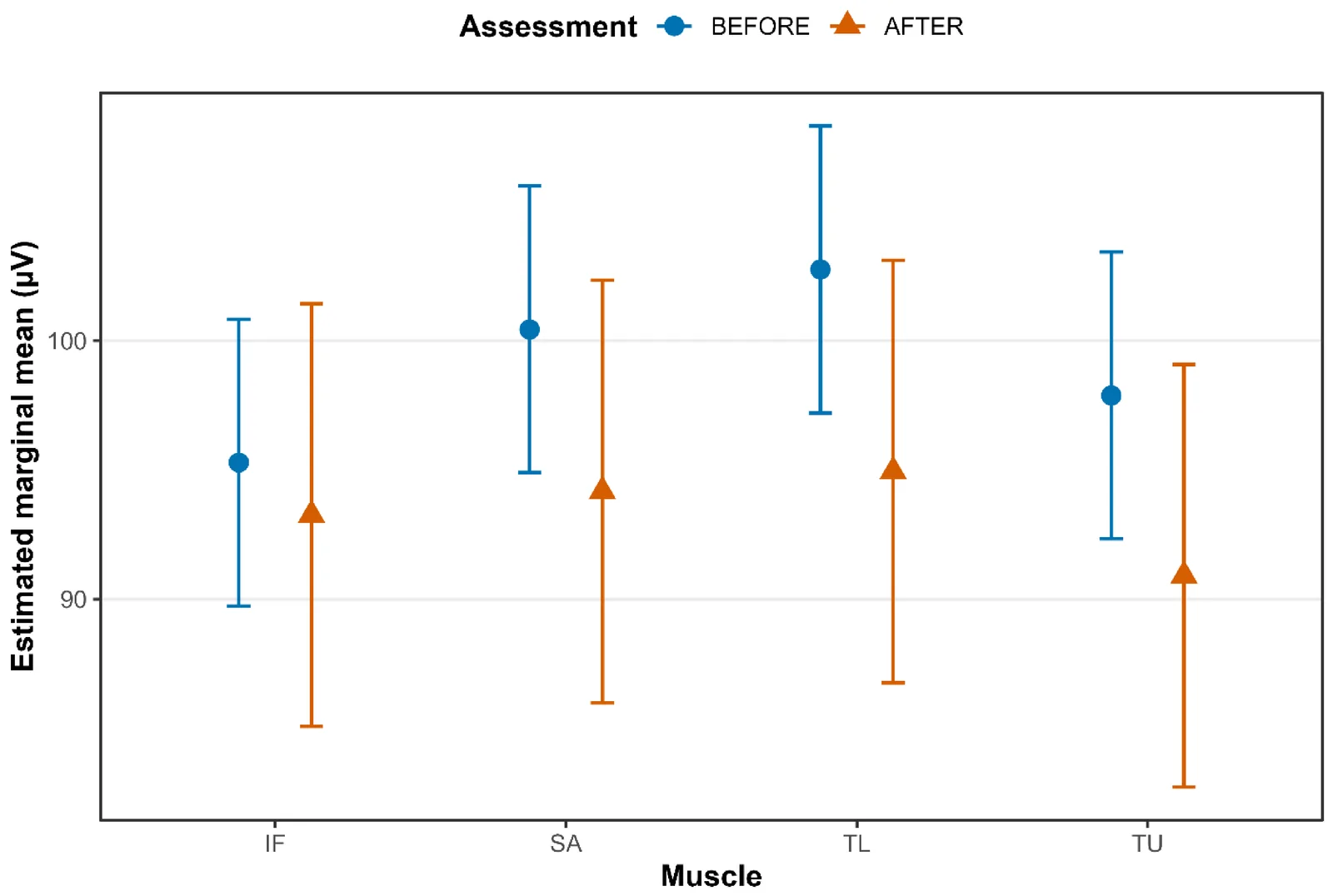
Model-estimated marginal means and 95% confidence intervals for maximum RMS amplitude recorded during muscle-specific maximal voluntary isometric effort tasks before (BEFORE) and after (AFTER) the volleyball training sessions. Estimates are presented for the infraspinatus (IF), serratus anterior (SA), lower trapezius (TL), and upper trapezius (TU) and were averaged equally across the three measurement days. The primary linear mixed-effects model included assessment condition, muscle, the assessment condition × muscle interaction, and measurement day as fixed effects, with correlated participant-specific random intercepts and assessment-condition slopes. Values are expressed in µV.

**Figure 3:**
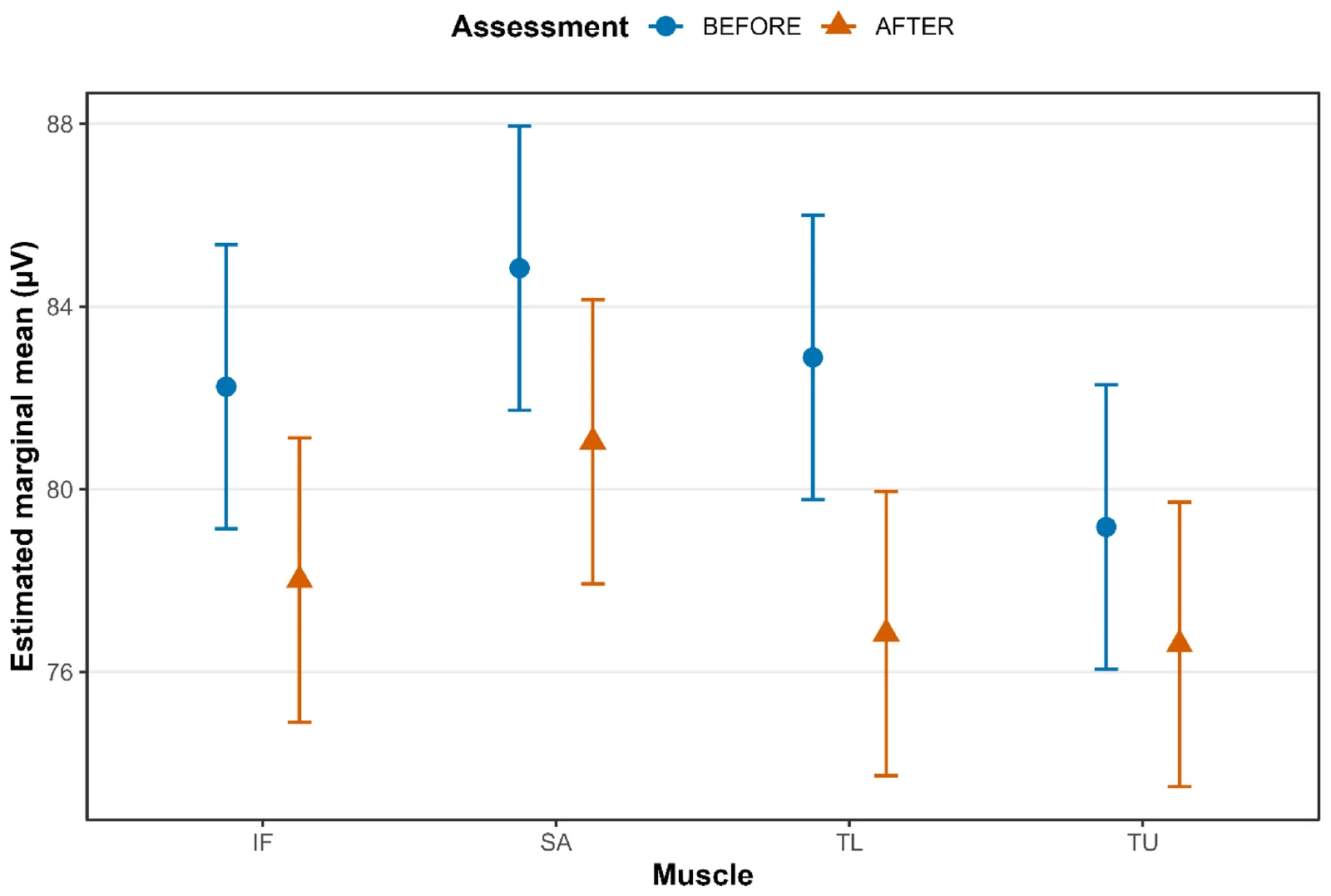
Model-estimated marginal means and 95% confidence intervals for mean RMS amplitude recorded during muscle-specific maximal voluntary isometric effort tasks before (BEFORE) and after (AFTER) the volleyball training sessions. Estimates are presented for the infraspinatus (IF), serratus anterior (SA), lower trapezius (TL), and upper trapezius (TU) and were averaged equally across the three measurement days. The primary linear mixed-effects model included assessment condition, muscle, the assessment condition × muscle interaction, and measurement day as fixed effects. Participant identity was included as a random intercept because both the correlated and uncorrelated assessment-condition random-slope specifications produced singular fits. Values are expressed in µV.

**Figure 4:**
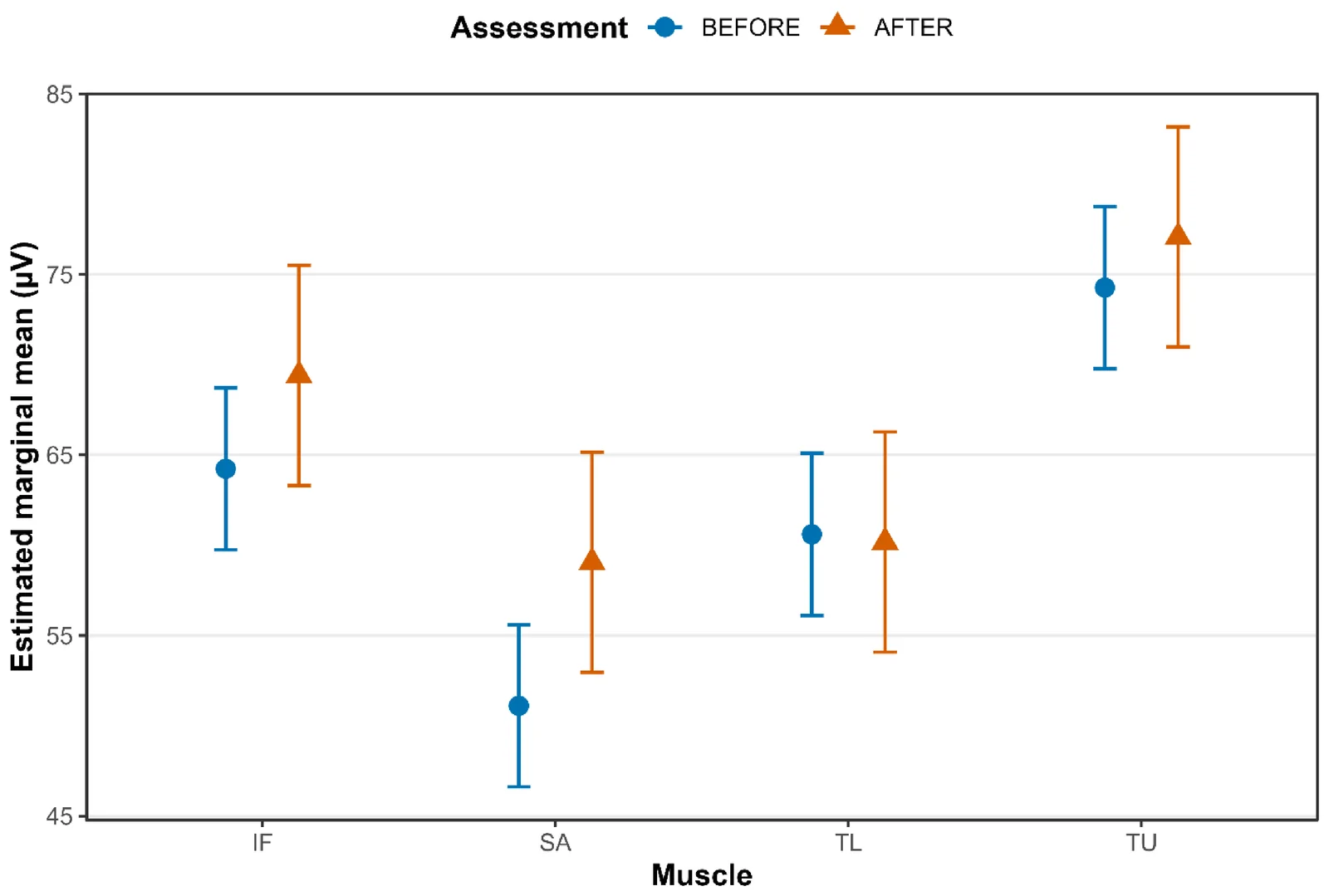
Model-estimated marginal means and 95% confidence intervals for median RMS amplitude recorded during muscle-specific maximal voluntary isometric effort tasks before (BEFORE) and after (AFTER) the volleyball training sessions. Estimates are presented for the infraspinatus (IF), serratus anterior (SA), lower trapezius (TL), and upper trapezius (TU) and were averaged equally across the three measurement days. The primary linear mixed-effects model included assessment condition, muscle, the assessment condition × muscle interaction, and measurement day as fixed effects, with correlated participant-specific random intercepts and assessment-condition slopes. Values are expressed in µV. Median RMS amplitude represents the median of the RMS amplitude observations and should not be confused with EMG median frequency.

**Figure 5:**
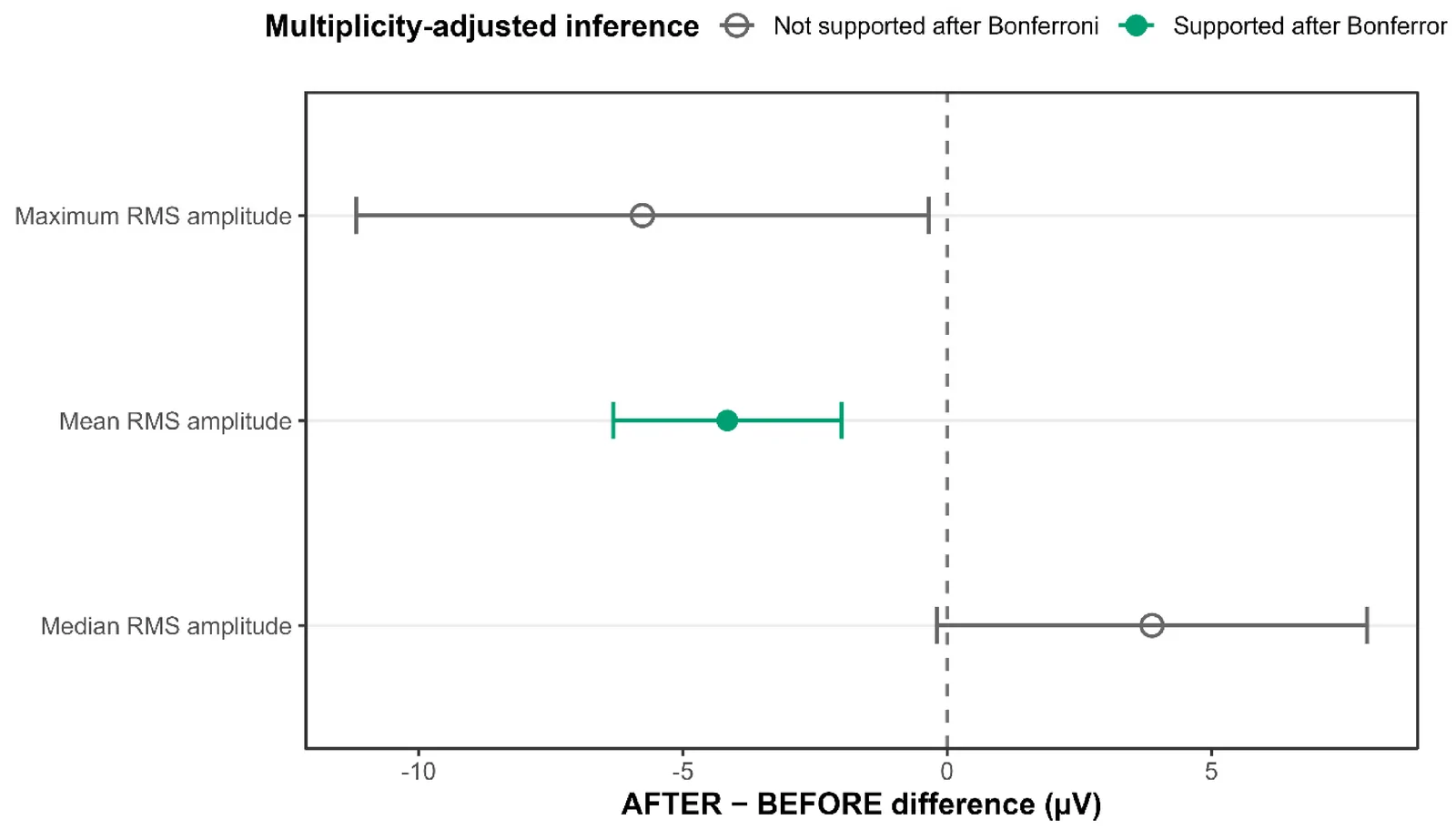
Model-estimated marginal AFTER–BEFORE contrasts for maximum, mean, and median RMS amplitudes. Points represent the estimated differences, and horizontal bars represent model-based 95% confidence intervals. Contrasts were obtained from the primary linear mixed-effects models and averaged equally across the four muscles and three measurement days. Negative values indicate lower RMS amplitude at the AFTER assessment, whereas positive values indicate higher amplitude. The vertical reference line denotes no difference. We applied a Bonferroni adjustment to *p*-values across the three EMG outcomes; only the mean RMS amplitude contrast remained statistically significant after adjustment. The displayed 95% confidence intervals are unadjusted.

Model-estimated marginal means by assessment condition and muscle are presented for maximum RMS amplitude in Figure 2, mean RMS amplitude in Figure 3, and median RMS amplitude in Figure 4. The corresponding overall marginal AFTER–BEFORE contrasts are summarized in Figure 5.

### 3.3. Sensitivity Analyses

The exploratory full three-way models produced marginal AFTER–BEFORE estimates that were essentially analogous to those from the primary models. Only the decrease in mean RMS amplitude remained statistically supported after Bonferroni adjustment in these models (Supplementary Table S2). Effect directions were also consistent across the primary, random-intercept-only, and exploratory full three-way specifications. However, the random-intercept-only models changed the multiplicity-adjusted inference for maximum and median RMS amplitudes, whereas the full three-way models agreed with the primary models. Mean RMS amplitude remained statistically supported under every specification (Supplementary Table S3). These findings indicate that the mean RMS result was robust, whereas the inferential interpretation of maximum and median RMS amplitudes depended on whether participant-specific condition slopes were represented.

**Figure 6:**
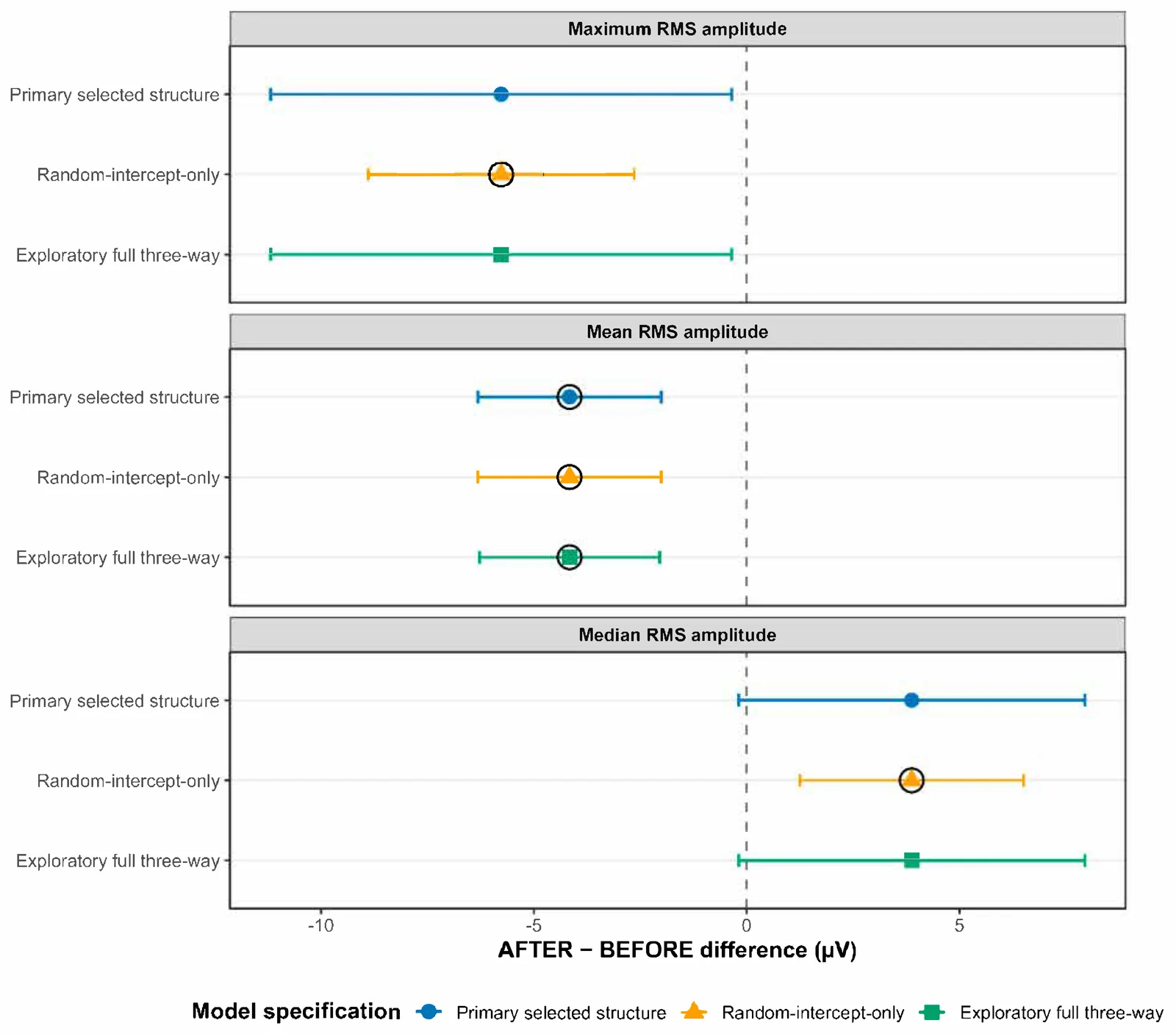
Sensitivity of the model-estimated marginal AFTER–BEFORE contrasts to alternative mixed-effects model specifications. For each EMG outcome, estimates and 95% confidence intervals are presented for the primary model with the selected participant-specific random-effects structure, the corresponding random-intercept-only model, and the exploratory full assessment condition × muscle × measurement day model. Negative values indicate lower RMS amplitude at the AFTER assessment, whereas positive values indicate higher amplitude. Effect directions were consistent across all model specifications. Mean RMS amplitude remained statistically significant after Bonferroni adjustment under every specification, whereas the adjusted inference for maximum and median RMS amplitudes changed under the random-intercept-only specification. We applied Bonferroni adjustment across the three EMG outcomes within each model specification. The displayed 95% confidence intervals are unadjusted.

Figure 6 illustrates the consistency of effect direction and the differences in multiplicity-adjusted inference across the primary, random-intercept-only, and exploratory full three-way model specifications, and Supplementary Table S3 reports these results numerically.

## 4. Discussion

The principal finding was a model-estimated BEFORE–AFTER decrease in mean RMS amplitude. This difference remained statistically supported after Bonferroni adjustment across the three RMS-amplitude outcomes and was directionally consistent across the evaluated model specifications. Maximum and median RMS amplitude did not show multiplicity-adjusted evidence of a BEFORE–AFTER difference in the primary models. Exploratory assessment condition × muscle tests did not provide statistically detectable evidence that the magnitude of the BEFORE–AFTER difference varied among muscles; however, the small participant-level sample precludes interpreting these nonsignificant interactions as evidence of physiological equivalence.

Both BEFORE and AFTER assessments were performed after completion of the standardized dynamic warm-up. The BEFORE assessment was obtained immediately after warm-up and before the subsequent volleyball-specific training components, whereas the AFTER assessment was obtained within 5–10 min after completion of the scheduled session. Thus, warm-up completion was standardized across the two assessments and did not constitute a difference in assessment status. Nevertheless, the AFTER assessment additionally incorporated the intervening volleyball-specific training exposure, elapsed time, repeated-testing order, and early recovery. In the absence of a non-training time-control condition, these components cannot be separated completely, and the observed AFTER–BEFORE difference should therefore be interpreted as a within-session change observed across the intervening training exposure rather than as definitive evidence of an isolated causal training effect.

Individual external and internal loads were also not quantified. Although the athletes completed the same scheduled team session, position-specific involvement and individual numbers of serves, attacks, blocks, jumps, and other high-load actions were unknown. Consequently, between-player variability in EMG-amplitude differences cannot be related to an individual training dose or used to infer readiness, adaptation, or appropriateness of the session load.

No statistically significant assessment condition × muscle interaction was identified for any outcome. Thus, the available data did not demonstrate different BEFORE–AFTER responses among the upper trapezius, lower trapezius, infraspinatus, and serratus anterior. These nonsignificant interactions should not be interpreted as evidence of physiological equivalence because the participant-level sample was limited and the EMG amplitudes were not normalized.

The sensitivity analyses reinforce the need for this cautious interpretation. Although the direction of every marginal contrast was unchanged across the evaluated model specifications, random-intercept-only models produced different multiplicity-adjusted conclusions for maximum and median RMS amplitudes. The exploratory full three-way models agreed with the primary random-slope models. Therefore, the decrease in mean RMS amplitude represents the most robust finding, whereas evidence concerning maximum and median RMS amplitudes was sensitive to the representation of participant-specific responses.

Controlled shoulder-fatigue studies in overhead athletes have reported changes in shoulder EMG amplitude or shoulder mechanics after standardized tasks [30,31,32]. Direct comparison with the present findings is limited because the current study evaluated the volleyball-specific portion of a scheduled team-training session without quantifying individual external or internal training load. The nonsignificant assessment condition × muscle interactions do not establish equivalent responses among the examined muscles.

The statistically supported decrease in mean RMS amplitude should therefore be interpreted only as a difference in one non-normalized amplitude-domain EMG summary measure between the post-warm-up BEFORE assessment and the post-session AFTER assessment. It does not establish reduced mechanical force, localized neuromuscular fatigue, readiness, training adaptation, or injury risk. Future studies should quantify individual external and internal training load, include an appropriate non-training or warm-up-only time-control condition, use normalized and spectral EMG measures, assess measurement reliability, and record mechanical force or joint torque where mechanical capacity is an intended outcome.

### Limitations and Future Directions

Several limitations affect the interpretation of the present findings. First, the study included only 16 athletes from a single university collegiate volleyball team, and no a priori sample-size calculation was performed. Although the repeated-measures design generated multiple observations per athlete, the number of independent experimental units remained 16. The study may therefore have had insufficient power to identify small or heterogeneous assessment condition × muscle, assessment condition × day, and higher-order interaction effects. Consequently, the absence of statistically significant interactions should be interpreted as an absence of statistically detectable evidence in this sample and not as proof that the muscles or measurement days were physiologically equivalent. The small and uneven distribution of athletes across playing positions also prevented reliable position-specific analyses.

Second, although both BEFORE and AFTER assessments were conducted after the standardized warm-up, the study did not include a non-training time-control or alternative-training condition. The BEFORE assessment was performed immediately after warm-up and before the subsequent volleyball-specific training components, whereas the AFTER assessment was performed within 5–10 min after completion of the scheduled session. The design therefore controlled the warm-up status of the two measurements more closely than a pre-warm-up versus post-session comparison would have done. Nevertheless, elapsed time, repeated testing, measurement order, the intervening volleyball-specific training exposure, and early recovery remain inseparable within the present design. Consequently, the AFTER–BEFORE difference cannot be regarded as definitive evidence of an isolated causal training effect.

Third, individual training load was not quantified using objective external-load or internal-load measures. Counts of serves, attacks, blocks and jumps, position-specific involvement, accelerometer- or player-tracking-derived load, session rating of perceived exertion, and validated subjective fatigue or recovery scores were not recorded. Because these data were not collected prospectively, subjective exertion and fatigue cannot be reconstructed retrospectively. Participation in the same team session therefore does not demonstrate that every athlete or shoulder muscle received an equivalent stimulus. Moreover, the absence of session-RPE or another validated self-report measure prevents assessment of whether the observed EMG-amplitude changes were accompanied by a subjectively perceived increase in exertion or fatigue. Future studies should combine EMG with direct measurements of force or joint torque with individual monitoring of overhead-action volume, movement intensity, session-RPE, and validated fatigue or recovery questionnaires.

Fourth, EMG amplitudes were expressed as absolute rather than normalized RMS values. This limits physiological comparisons among muscles, participants, and weekly measurements because absolute EMG amplitude is influenced by electrode position, skin–electrode impedance, subcutaneous tissue, muscle architecture, and recording geometry. The same electrodes remained in situ between the BEFORE and AFTER recordings within each measurement day, but perspiration, movement, clothing contact, and changes in recording conditions during the intervening training period could have affected the signal. Between weekly sessions, electrodes were reapplied using the same anatomical landmarks, but persistent skin markings, photographic coordinate templates, and formal impedance measurements were not used. Therefore, both within-session changes in recording conditions and between-week electrode-repositioning variability may have contributed to the observed amplitude differences. Absolute between-muscle differences cannot establish that one muscle was more strongly activated or more fatigued than another, and nonsignificant assessment condition × muscle interactions do not demonstrate physiological equivalence.

Fifth, the study evaluated mean and median RMS amplitude but did not include spectral EMG indices, such as median or mean frequency. The recorded amplitude changes therefore describe changes in the EMG signal and should not be regarded as direct evidence of peripheral fatigue. Intra-session reliability of the maximal trials was also not estimated, limiting the ability to separate physiological change from trial-to-trial measurement variability. In addition, the models contained multiple interaction terms relative to the number of independent participants; higher-order estimates should therefore be regarded as exploratory, particularly when they were isolated and not supported by a consistent pattern across outcomes.

Finally, neuromuscular function was assessed during a standardized isometric task, whereas volleyball involves dynamic, high-velocity and multiplanar actions dependent on whole-body kinetic-chain coordination. The test may therefore have had limited sensitivity to acute neuromuscular changes arising during sport-specific serves, attacks and blocking actions. Future investigations should use larger samples, prospectively defined sample-size calculations, normalized and spectral EMG measures, formal reliability assessment, quantified individual training load, comparable pre- and post-exercise testing conditions, appropriate control conditions and dynamic volleyball-specific tasks.

## 5. Conclusions

In this exploratory sample of male collegiate volleyball players, mean RMS amplitude differed between an assessment performed immediately after a standardized warm-up and before the subsequent volleyball-specific training components and an assessment performed within 5–10 min after completion of the scheduled team-training session, whereas maximum and median RMS amplitudes did not show multiplicity-adjusted BEFORE–AFTER differences. Exploratory assessment condition × muscle analyses did not provide statistically detectable evidence that the magnitude of the BEFORE–AFTER difference varied among the four muscles, but the limited sample does not establish equivalent responses. Because both assessments followed the standardized warm-up, warm-up status was held constant with respect to completion of the warm-up. However, the AFTER assessment additionally followed the intervening volleyball-specific training exposure, elapsed time, and early recovery. These data do not establish localized neuromuscular fatigue, altered mechanical capacity, training adaptation, athlete readiness, or injury risk.

## Figures and Tables

**Table 1:** Descriptive EMG amplitude values pooled across three weekly assessments in 16 participants.

Outcome	Assessment	Muscle	Observations, n	Participants, n	Mean, µV	SD, µV
Maximum RMS amplitude	Before	IF	48	16	95.28	18.1
		SA			100.43	11.84
		TL			102.75	18.69
		TU			97.88	12.54
	After	IF	48	16	93.26	20.79
		SA			94.17	24.37
		TL			94.94	19.3
		TU			90.91	17.56
Mean RMS amplitude	Before	IF	48	16	82.25	10.35
		SA			84.84	15.09
		TL			82.89	12.2
		TU			79.18	12.01
	After	IF	48	16	78.02	12.07
		SA			81.04	5.23
		TL			76.84	8.73
		TU			76.61	7.57
Median RMS amplitude	Before	IF	48	16	64.24	12.62
		SA			51.1	11.88
		TL			60.6	10.29
		TU			74.27	16.04
	After	IF	48	16	69.41	17.79
		SA			59.06	24.06
		TL			60.18	6.35
		TU			77.08	14

Values are descriptive means and standard deviations calculated from up to 48 repeated observations per muscle–condition combination, comprising 16 participants assessed on three measurement days. These observations are not independent; inferential estimates and confidence intervals are therefore reported from the mixed-effects models rather than from the pooled descriptive data: TU, upper trapezius; TL, lower trapezius; IF, infraspinatus; SA, serratus anterior.

**Table 2:** Type III tests of fixed effects from the primary linear mixed-effects models for the three surface-EMG amplitude outcomes.

Outcome	Fixed Effect	F(df1, df2)	*p*-Value	Partial η^2^
Maximum RMS amplitude	Assessment (BEFORE vs. AFTER)	5.15(1, 15)	0.038	0.255
	Muscle	2.16(3, 344)	0.093	0.018
	Measurement day	3.16(2, 344)	0.044	0.018
	Assessment × muscle	0.705(3, 344)	0.549	0.006
Mean RMS amplitude	Assessment (BEFORE vs. AFTER)	14.427(1, 359)	<0.001	0.038
	Muscle	3.601(3, 359)	0.014	0.029
	Measurement day	1.196(2, 359)	0.303	0.006
	Assessment × muscle	0.431(3, 359)	0.731	0.003
Median RMS amplitude	Assessment (BEFORE vs. AFTER)	4.126(1, 15)	0.06	0.215
	Muscle	46.151(3, 344)	<0.001	0.286
	Measurement day	8.294(2, 344)	<0.001	0.046
	Assessment × muscle	1.855(3, 344)	0.137	0.015

Assessment condition comprised the BEFORE and AFTER measurements. Measurement day was included as a categorical adjustment factor. We used participant-specific correlated random intercepts and assessment-condition slopes for maximum and median RMS amplitudes. We used a random-intercept model for mean RMS amplitude because both random-slope candidates produced singular fits. We used Type III tests with sum-to-zero contrasts and Satterthwaite-adjusted degrees of freedom. The *p*-values in this table are unadjusted; multiplicity-adjusted marginal AFTER–BEFORE contrasts are reported in Supplementary Table S2. Model R^2^ values and random-effects structures are reported in the text and Supplementary Table S1.

## Data Availability

The data supporting the findings of this study are available from the corresponding author upon reasonable request. The data are not publicly available because they contain information derived from human participants and their distribution is subject to applicable ethical and confidentiality considerations.

## Supplementary Materials

The following supporting information can be downloaded at https://www.mdpi.com/article/10.3390/jcm15166460/s1. Supplementary Table S1. Random-effects structure, model fit, and convergence diagnostics for the primary linear mixed-effects models. Supplementary Table S2. Model-estimated marginal AFTER–BEFORE contrasts for the three surface-EMG amplitude outcomes. Supplementary Table S3. Sensitivity analysis of marginal AFTER–BEFORE contrasts across alternative mixed-effects model specifications.
