# Supplementary material for: Surface-EMG Amplitude Differences Before and After Scheduled University Team Training Following a Standardized Warm-Up in Male Collegiate Volleyball Players
Source: J Clin Med. 2026 Aug 20;15(16):6460. doi: 10.3390/jcm15166460 (PMC13513708; doi:10.3390/jcm15166460)
Supplement: Supplementary file 1 [file jcm-15-06460-s001.zip › jcm-4449721-supplementary.pdf]

Supplementary table legend

Supplementary Table S1. Random-effects structure, model fit, and convergence diagnostics for the primary linear mixed-effects models.

Primary model: EMG outcome ~ assessment condition × muscle + measurement day + selected participant-specific random structure

Outcome	Selected random structure	Converged	Singular fit	Intercept variance	Condition-slope variance	Intercept–slope correlation	Residual variance	Marginal R^2	Conditional R^2
**Maximum RMS amplitude**	Correlated random slope	Yes	No	95.132	65.734	0.818	225.714	0.051	0.332
**Mean RMS amplitude**	Random intercept	Yes	No	1.574	—	—	115.203	0.069	0.082
**Median RMS amplitude**	Correlated random slope	Yes	No	49.235	31.003	0.792	163.512	0.254	0.426

Notes:

Converged and Singular fit refer to the selected primary model. The random-slope term represents participant-specific variation in the BEFORE–AFTER assessment contrast.

For mean RMS amplitude, correlated and uncorrelated random-slope candidates were singular; the non-singular random-intercept fallback was selected.

An em dash (—) indicates that a condition-slope variance or intercept–slope correlation was not estimated. R^2 values are reported as marginal (fixed effects) and conditional (fixed and random effects).

Supplementary Table S2

Model-estimated marginal AFTER–BEFORE contrasts for the three surface-EMG amplitude outcomes.

Outcome	AFTER − BEFORE estimate, µV	SE. µV	df	95% CI lower, µV	95% CI upper, µV	t ratio	Unadjusted p-value	Bonferroni-adjusted p-value	Supported after Bonferroni adjustment
Maximum RMS amplitude	-5.77	2.54	15.0	-11.19	-0.35	-2.27	0.038	0.115	No
Mean RMS amplitude	-4.16	1.08	345.0	-6.28	-2.04	-3.87	<0.001	<0.001	Yes
Median RMS amplitude	3.88	1.91	15.0	-0.19	7.94	2.03	0.060	0.181	No

Notes. Estimates are marginal contrasts from exploratory full three-way linear mixed-effects models containing assessment condition × muscle × measurement day. Negative estimates indicate lower RMS amplitude AFTER than BEFORE. Exact p-values are retained in the Source data sheet; values below 0.001 are displayed as $p < 0.001$. We applied a Bonferroni adjustment across the three EMG outcomes (adjusted $\alpha = 0.0167$). These sensitivity estimates complement, but do not replace, the primary-model contrasts reported in main-manuscript Supplementary Table 2.

Source file: Sensitivity_full_three_way_condition_contrasts.csv

Supplementary Table S3

Sensitivity analysis of marginal AFTER–BEFORE contrasts across alternative mixed-effects model specifications

Outcome	Model specification	AFTER – BEFORE estimate, µV	95% CI lower, µV	95% CI upper, µV	Bonferroni-adjusted p-value	Supported after Bonferroni adjustment	Direction consistent with primary model	Inference consistent with primary model
Maximum RMS amplitude	Primary parsimonious model	-5.77	-11.19	-0.35	0.115	No	Reference	Reference
Maximum RMS amplitude	Random-intercept-only model	-5.77	-8.89	-2.64	<0.001	Yes	Yes	No
Maximum RMS amplitude	Exploratory full three-way model	-5.77	-11.19	-0.35	0.115	No	Yes	Yes
Mean RMS amplitude	Primary parsimonious model	-4.16	-6.32	-2.01	<0.001	Yes	Reference	Reference
Mean RMS amplitude	Random-intercept-only model	-4.16	-6.32	-2.01	<0.001	Yes	Yes	Yes
Mean RMS amplitude	Exploratory full three-way model	-4.16	-6.28	-2.04	<0.001	Yes	Yes	Yes
Median RMS amplitude	Primary parsimonious model	3.88	-0.19	7.94	0.181	No	Reference	Reference
Median RMS amplitude	Random-intercept-only model	3.88	1.25	6.50	0.012	Yes	Yes	No
Median RMS amplitude	Exploratory full three-way model	3.88	-0.19	7.94	0.181	No	Yes	Yes

Notes. Estimates are model-estimated marginal AFTER–BEFORE contrasts averaged over muscles and measurement days. Negative estimates indicate lower RMS amplitude AFTER than BEFORE. We applied a Bonferroni adjustment across the three EMG outcomes (adjusted $\alpha = 0.0167$). The primary parsimonious model included assessment condition × muscle and measurement day with the participant-specific random structure selected after convergence and singularity checks. The random-intercept-only model replaced that structure with a participant-intercept-only structure. The exploratory full model included assessment condition × muscle × measurement day. Direction and inference columns compare each alternative specification with the primary model; 'Reference' identifies the primary model. Inference consistency denotes agreement in whether the Bonferroni-adjusted p-value crossed $\alpha = 0.0167$. Exact numerical values are retained in the Source data sheet.

Source file: Sensitivity_robustness_comparison.csv
